# An improved string composition method for sequence comparison

**DOI:** 10.1186/1471-2105-9-S6-S15

**Published:** 2008-05-28

**Authors:** Guoqing Lu, Shunpu Zhang, Xiang Fang

**Affiliations:** 1 Department of Biology, University of Nebraska, Omaha, NE 68182, USA; 2 Department of Statistics, University of Nebraska, Lincoln, NE 68583, USA

## Abstract

**Background:**

Historically, two categories of computational algorithms (alignment-based and alignment-free) have been applied to sequence comparison–one of the most fundamental issues in bioinformatics. Multiple sequence alignment, although dominantly used by biologists, possesses both fundamental as well as computational limitations. Consequently, alignment-free methods have been explored as important alternatives in estimating sequence similarity. Of the alignment-free methods, the string composition vector (CV) methods, which use the frequencies of nucleotide or amino acid strings to represent sequence information, show promising results in genome sequence comparison of prokaryotes. The existing CV-based methods, however, suffer certain statistical problems, thereby underestimating the amount of evolutionary information in genetic sequences.

**Results:**

We show that the existing string composition based methods have two problems, one related to the Markov model assumption and the other associated with the denominator of the frequency normalization equation. We propose an improved complete composition vector method under the assumption of a uniform and independent model to estimate sequence information contributing to selection for sequence comparison. Phylogenetic analyses using both simulated and experimental data sets demonstrate that our new method is more robust compared with existing counterparts and comparable in robustness with alignment-based methods.

**Conclusion:**

We observed two problems existing in the currently used string composition methods and proposed a new robust method for the estimation of evolutionary information of genetic sequences. In addition, we discussed that it might not be necessary to use relatively long strings to build a complete composition vector (CCV), due to the overlapping nature of vector strings with a variable length. We suggested a practical approach for the choice of an optimal string length to construct the CCV.

## Background

The increasing proliferation of biological sequence data has created tremendous opportunities for biologists and medical researchers to address both fundamental issues (e.g., molecular evolution) and practical problems (e.g., drug design). On the other hand, it poses many computational challenges for theoretical scientists to create efficient and reliable methods or algorithms for sequence analyses and knowledge mining. Sequence comparison, an essential operation for gene finding and protein function annotation, is one such challenge. The methods for sequence comparison are classified into two categories, alignment-based and alignment-free. The alignment-based sequence analysis methods have both fundamental and computational limitations [1-4]. For example, these methods cannot deal with changes like chromosome reversal or gene translocation. They also encounter difficulties in aligning dissimilar sequences. Another drawback with sequence alignment is its computational complexity, where no optimal solution can be achieved when a large number of sequences are compared. Consequently, considerable efforts have been made to seek for alternative, i.e., alignment-free, methods for sequence comparison.

The alignment-free methods seen in the past few decades can be divided into three categories: gene contents [5-7], data compression [8-11], and string (or word) composition [12-18]. Of these methods, the string-composition-based methods, especially the composition vector (CV) method [12] and the complete composition vector (CCV) method [16], have received substantial attention. The CV method uses strings of a fixed length whereas the CCV method uses strings of multiple lengths. The CCV method was found to provide finer evolutionary information than the CV method; however, it has disadvantages regarding computing time and memory usage. Both of the above mentioned methods apply a Markov model assumption to estimate the random background of observed frequencies, which has been found to be problematic, as detailed in Section 2. In this paper, we will provide an improved CCV (ICCV) method and demonstrate that this new method is more robust and efficient in performing sequence comparison compared with the existing CCV method. The issue of how to build a more informative CCV, i.e., how to select the maximum vector string length for better evolutionary information representation, will be addressed as well.

The contents of this paper are arranged as follows. In the Methods section, we point out the two aforementioned problems in the existing CV or CCV methods and describe our new ICCV method. In the Results section, we compare the CCV and ICCV methods through simulations and experimental data analysis. In the Discussion section, we discuss the potential impact of the simple assumption of a uniform and independent model and issues related to selecting the maximum string length for CCV construction.

## Methods

### Existing CV and CCV methods

Define *S *as a DNA sequence consisting of *N *nucleotides. Let *f*(*α*_1_...*α*_*k*_) be the observed frequency of the *k*-mer string *α*_1_...*α*_*k*_, where *α*_*i *_is one of the four nucleotides A, C, T, or G and *k *is the string length (1 ≤ *k *<*N*). We define $S_{k}=(f_{1},f_{2},...,f_{4^{k}})$ as a vector of observed frequencies for a given *k*, where 4^*k *^is the number of *k*-mer strings, and let *γ*_*K *_= (*S*_1_, *S*_2_, ..., *S*_*K*_) as a combined vector for some constant *K *(*K *<* N*), where *K *is the maximum string length considered. From the perspective of molecular evolution, *S*_*k *_or *γ*_*K *_reflects both random mutation and selection, and the random background needs to be normalized in order to represent genetic information contributed by natural selection. After the normalization of observed frequencies, *S*_*k *_is converted into a composition vector (CV), and *γ*_*K *_is transformed into a complete composition vector (CCV).

The method to normalize the observed frequencies of different *k*-mer strings in *S *was originally proposed by Brendel et al. [20] and has been used with minor modifications for phylogenetic studies of prokaryotes and viruses [12,16]. We have found two problems associated with string frequency normalization in existing methods. To explicate these problems, we reiterate the normalization equation of the observed frequency of *α*_1_...*α*_*k*_, i.e., *f*(*α*_1_...*α*_*k*_), described in [12] as below:

(1) $$a(\alpha_{1}\cdots \alpha_{k})=\frac{f(\alpha_{1}\cdots \alpha_{k})-f^{0}(\alpha_{1}\cdots \alpha_{k})}{f^{0}(\alpha_{1}\cdots \alpha_{k})},$$

where $f^{0}(\alpha_{1}\cdots \alpha_{k})=\frac{f(\alpha_{1}\cdots \alpha_{k-1})f(\alpha_{2}\cdots \alpha_{k})}{f(\alpha_{2}\cdots \alpha_{k-1})}.\frac{\left(N-k+1)(N-k+3\right)}{{\left(N-k+2\right)}^{2}}$ for *k *≥ 3.

**Figure 1:**
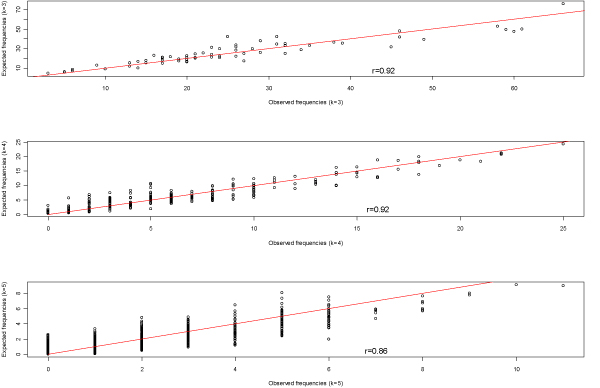
Correlation between the observed frequencies and the estimated expected frequencies of strings with length *k *= 3, 4, 5, respectively, in a randomly selected sequence from our database. Reference lines in the plots designate *y *= *x*.

First, there is a positive correlation between the observed frequency *f*(*α*_1_...*α*_*k*_) and the estimated expected frequency *f*^0^(*α*_1_...*α*_*k*_). We computed both quantities for *k *= 3, 4, 5 using a randomly chosen virus sequence. The correlation coefficients between *f*^0^(*α*_1_...*α*_*k*_) and *f*(*α*_1_...3;*α*_*k*_) are 0.92, 0.92 and 0.86, respectively, for *k *= 3, 4, 5 with *p *< 0.0001 (Fig. 1).

Define *E*_0_[*f*(*α*_1_...*α*_*k*_)] as the true expected frequency of *k*-mer string *α*_1_...*α*_*k *_in *S*. Since there exists a highly positive correlation between *f*^0^(*α*_1_...*α*_*k*_) and *f*(*α*_1_...*α*_*k*_), the difference between them tends to be smaller than the difference between *f*(*α*_1_...*α*_*k*_) and *E*_0_[*f*(*α*_1_...*α*_*k*_)], indicating the information contributed by selective evolution is underestimated.

Another problem associated with Eq. [1] is the denominator. As originally proposed in [20], a square root needs to be applied to the denominator. Without such an operation, the normalized frequency tends to be over-standardized.

### Improved CCV (ICCV) method

We assume that the four bases A, C, T, and G occur randomly with equal chance and derive the expected frequency of a *k*-mer string and the frequency variance in a given sequence *S *based upon this simple assumption. Define *x*_*i *_as follows:

$$x_{i}=\{\begin{array}{l} 1,\text{if the }k\text{-mer string begins at position }i\\ 0,\text{otherwise} \end{array},$$

where *i *= 1, 2, 3, ..., *N *- *k *+ 1 and *N *- *k *+ 1 is the maximum frequency one can observe for string *α*_1_...*α*_*k *_in DNA sequence *S *of length *N*. Therefore, it can be shown that $f(\alpha_{1}...\alpha_{k})=\sum_{i=1}^{N-k+1}x_{i}$. The expectation and variance of *f*(*α*_1_⋯*α*_*k*_) are given as

$$E[f(\alpha_{1}\cdots \alpha_{k})]=\sum_{i=1}^{N-k+1}E(x_{i})=\frac{N-k+1}{4^{k}},$$

and

$$Var[f(\alpha_{1}\cdots \alpha_{k})]=\frac{\left(N-k+1\right)}{4^{k}}(1-\frac{1}{4^{k}})-\frac{2}{4^{2k}}(k-1)(N-\frac{3}{2}k+1)+\frac{2}{4^{k}}\sum_{t=1}^{k-1}(N-k+1-t)\frac{J_{t}}{4^{t}},$$

where $J_{t}=\{\begin{array}{l} 1,\;\;\text{if (}a_{1}...a_{k-t}\text{)=(}a_{t+1}...a_{k}\\ 0,\;\;\text{otherwise} \end{array}$, for *t *= 1, 2, 3,..., *k *- 1. For a full derivation of the above equation, readers may refer to [21,22].

With both expectation and variance derived, the normalization function for the observed frequency of a *k*-mer string is given as:

(2) $$\frac{f(\alpha_{1}\cdot \cdot \cdot \alpha_{k})-E[f(\alpha_{1}\cdot \cdot \cdot \alpha_{k})]}{\sqrt{Var[f(\alpha_{1}\cdot \cdot \cdot \alpha_{k})]}},$$

for *k *≥ 1.

We construct an improved CCV (ICCV) with the normalized frequencies of all *k*-mer strings computed using Eq. [2]. Since *E*[*f*(*α*_1_...*α*_*k*_)] is a theoretical value based on *N *and *k*, it is independent of *f*(*α*_1_...*α*_*k*_) for a fixed *k*. Therefore, the ICCV method we proposed does not experience the underestimation problem of the existing CCV methods. Another advantage of ICCV over CCV is that ICCV is constructed for any *k *but CCV is constructed for *k *> 3. The latter neglects the evolutionary information contained in *1*-mer and *2*-mer strings.

### Distance measurement

Let *α *= (*a*_1_, *a*_2_, ..., *a*_*T*_) and β = (*b*_1_, *b*_2_, ..., *b*_*T*_) be the CCV or the ICCV of two DNA sequences *A *and *B*, respectively. To calculate *D*(*A*, *B*), the distance between *A *and *B*, we adopt a distance measurement in this paper as detailed below:

$$D(A,B)=\frac{1-C(\alpha ,\beta )}{2},$$

where $C(\alpha ,\beta )=\frac{\sum_{i=1}^{T}a_{i}\times b_{i}}{(\sum_{i=1}^{T}a_{i}^{2}\times \sum_{i=1}^{T}b_{i}^{2}{)}^{1/2}}$. *C*(*α*, *β*) is the cosine of the angle between *α *and *β*.

### Data sets

**Figure 2:**
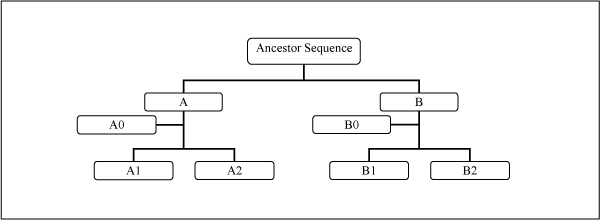
The predefined tree topology used to generate nucleotide sequences for the simulation study.

To generate simulation data sets to compare the performance of the ICCV and the CCV methods, we adopted a similar approach as in [8]. In brief, an ancestor sequence was randomly picked from our influenza virus database; and the progeny sequences were derived through simulation using different types of mutations (insertion, deletion, substitution, inversion, transposition or translocation) and following a pre-defined tree topology (Fig. 2). Six types of mutations at the rate of 9–15% were applied to generate A1 and A2 from A, and B1 and B2 from B. Three types of mutations (insertion, deletion, substitution) at the rate of 2–5% were used to generate A0 from A and B0 from B. A total of 1000 data sets were generated for phylogenetic analysis.

Besides the simulated data sets, we used a real dataset to compare the ICCV and the CCV methods. Fifty-four influenza A viral HA sequences were used. Each has approximately 1,659 base pairs. Based upon alignment-based phylogenetic analyses, each sequence was assigned a clade number by the International H5N1 Evolution Working Group (RO Donis, personal communication) [23].

### Data analysis and visualization

Statistical package R version 2.5.1 was used for programming and implementation of the CCV and ICCV methods. The trees were generated using the Neighbor-joining program in the PHYLIP 3.6.4 package. The resulting phylogenetic trees were displayed with MEGA 4.

## Results

### Analysis of simulation data sets

**Figure 3:**
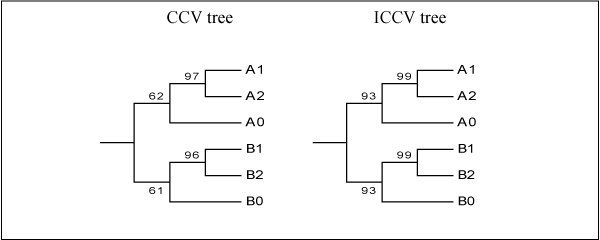
Consensus trees of simulated sequences constructed based on the CCV and ICCV methods for *K *= 6.

Both CCV and ICCV trees for *K *= 6 show the same topology of six sequences, as shown in Fig. 3. However, the ICCV tree provides much higher bootstrapping values in support of two major clades. This indicates the ICCV method is more robust in resolving phylogenetic relationships of remotely related clades than the existing CCV method.

### Application on influenza A virus lineage Analysis

**Figure 4:**
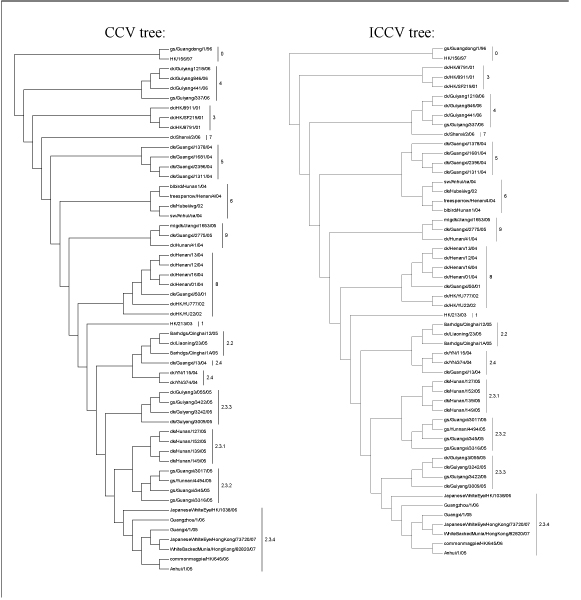
Phylogenetic trees obtained from the experimental sequence set using the CCV and ICCV methods for *K *= 7.

As shown in Fig. 4, ICCV and CCV trees for *K *= 7 agree with each other in the clade designation (denoted as 0, 1, ..., 9), but at the sub-clade level it appears that the designation based on the ICCV method is more convincing. For example, it is logical to assign the viral strain dk/Guangxi/13/4 to Sub-clade 2.4 as shown in the ICCV tree. However, this is not the case when examining the CCV tree. In addition, the positions of Clade 3, Clade 7 and Sub-clade 2.3.3 on the ICCV tree are not the same as on the CCV tree. When comparing trees generated from different methods, both the ICCV tree and the tree constructed by the H5N1 Working Group have exactly the same topology, which suggests that the ICCV method is more dependable than the existing CCV method.

## Discussion

### Does the uniform and independent assumption matter?

As we can envision, the only potential weakness associated with the ICCV method is the assumption of a uniform and independent model. It has been shown that the null hypothesis of equiprobable occurrence of different nucleotides is reasonable in the context of the DNA structures that have evolved from a "primordial soup' or 'base pool' containing equal quantities of each base [21]. Sege and Saxberg (1982) [24] have discussed this issue thoroughly. The hypothesis of independent occurrence of different nucleotides has also been accepted in numerous situations, particularly in the analysis of relatively short strings [21]. Arritia et al. [25] showed that the approximation of actual dependence in a DNA sequence to the theory of independence of bases is quite good.

We used our influenza H5N1 virus sequence database to examine the assumptions of uniformity and independence. Chi-square tests reject that the four nucleotides A, C, T, and G occur in equal probabilites (*p *< 0.0001) or occur independently of one another (*p *< 0.0001). Although the assumption does not generally hold, both results from the analyses of simulated data and experimental data showed that our improved method is more robust than the existing CCV method, indicating that the violation of the assumption on base composition has no significant impact on the accuracy of the ICCV method.

### Is increasing the maximum string length necessary?

**Figure 5:**
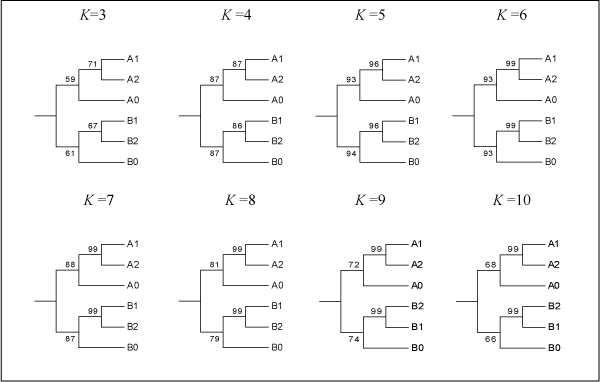
Consensus trees obtained from simulated sequences using the ICCV method for *K *= 3, 4,..., 10, respectively.

Wu et al. (2007) [19] suggested that increasing the maximum string length results in a vector containing finer evolutionary information. To investigate this issue, we used the same simulated sequences data as in section 3.1, and constructed the ICCV trees for *K *= 3, 4, 5, 7, 8, 9, 10 (Fig. 5). For the purpose of comparison, we also show the ICCV tree for *K *= 6 from Fig. 3. In Fig. 5, it is clearly shown that as *K *increases from 3 to 5, the supporting values significantly improve. However, this trend declines as *K *increases from 6 to 10. Obviously, in this case, *K *= 5 or 6 is a cutoff point, which means increasing *K *after a certain number may not necessarily improve the result. Therefore, it might not be the case that increasing the maximum string length would result in a vector containing finer evolutionary information.

The reason for this is that the overlapping nature of strings with multiple lengths causes the overlap of evolutionary information carried by each individual CV. As multiple CVs are combined into a complete CV, the complete CV collects the exclusive evolutionary information that each CV contains, but at the same time the overlapping information that individual CVs contain is also summed up. Therefore, increasing the string length *K *to a certain point will certainly improve the result, but the trend of improvement reaches its peak and afterwards declines. The question is how to choose an optimal string length for construction of the CCV, which will be discussed next.

### How to choose an optimal string length for the CCV

Firstly, all the DNA sequences in the dataset are concatenated into a single sequence *W *of length *M*, which provides an empirical nucleotide distribution for the class of sequences in the dataset. Then *S*_*k *_for *W *is computed. Since *S*_*k *_is the vector of observed frequencies of all the *k*-mer strings in *W*, $\frac{S_{k}}{M-k+1}=(q_{k(1)},q_{\left(k2\right)},...,q_{k(4^{k})})$ is the observed probability for all the *k*-mer strings. As for a random sequence, the probability for all the *k*-mer strings is $p=(p_{1},p_{2},...,p_{4^{k}})$, where $p_{i}=\frac{1}{4^{k}}$ for i = 1, 2,...., 4^*k*^. Therefore, we can determine the difference between these two probability distributions by their Kullback-Leibler distance:

$$D_{k}(W)=\sum_{i=1}^{4^{k}}q_{k(i)}\log_{2}(\frac{q_{k(i)}}{p_{i}}).$$

*D*_*k*_(*W*) would be small if the two distributions are close to each other, which indicates that *S*_*k *_does not contain rich evolutionary information and should be excluded from calculating the ICCVs.

**Figure 6:**
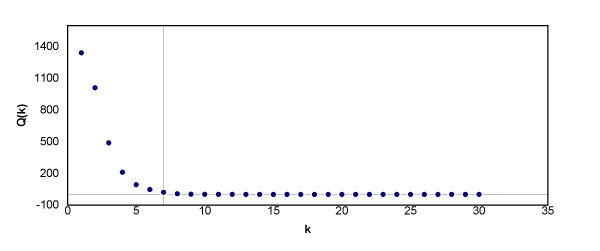
*Q*(*k*) = *D*_*k*_(*A*)/*D*_*k*_(*B*) for *k *= 1, 2,..., 30. The two reference lines designate *k *= 7 and *Q*(*k*) = 1, respectively.

To apply the above method to the experimental dataset in Section 3.2, we concatenated all 54 sequences in the experimental dataset into sequence *A *and calculated *D*_*k*_(*A*) for *k *= 1, 2, ..., 30. Similarly, we calculated *D*_*k*_(*B*) for *k *= 1, 2, ..., 30, where *B *is a randomly generated sequence with the same length as sequence *A*. Then we computed $Q(k)=\frac{D_{k}(A)}{D_{k}(B)}$ for *k *= 1, 2, ...., 30 (Fig. 6). In Fig. 6, we can see that the magnitude of *Q*(*k*) is fairly large when *k *is small. As *k *increases, *Q*(*k*) starts to decrease, and then it reaches a steady state at *Q*(*k*) = 1 when *k *is larger than 7. The reason for this is that the effect of selective evolution is more significant on shorter strings than it is on longer strings. Therefore, *D*_*k*_(*A*) is much larger than *D*_*k*_(*B*) when *k *is small. However, as *k *increases, the effect of selective evolution on *k*-mer strings starts to decline. Thus, the behavior of *k*-mer strings in sequence *A *becomes more similar to that of a random sequence and *D*_*k*_(*A*) becomes closer to *D*_*k*_(*B*), which indicates that less evolutionary information is carried by *S*_*k*_. For the experimental dataset, since *Q*(*k*) is fairly close to 1 when *k *is larger than 7, an appropriate choice for the maximum (optimal) string length *K *would be 7.

## Conclusion

In this paper, we show that the existing CV and CCV methods underestimate the evolutionary information contained in a DNA sequence due to the Markov model assumption and the denominator used for the normalization of observed string frequencies. Experiments using simulated and experimental data sets demonstrated that our ICCV method generates more accurate and robust results compared with the currently used CCV method. The consistency between the ICCV tree and the alignment-based tree recommended by the International H5N1 Evolution Working Group indicates that the ICCV method is a valuable alternative to the alignment-based methods. It is also shown that the violation of the assumption about base composition has no significant impact on the accuracy of the ICCV method. As to the issue related to maximum string length, we believe that it is not necessary to use relatively long strings to construct the CCV due to the overlapping nature of strings with variable length. We suggest a practical approach for choosing the optimal string length for the CCV.

## Competing interests

The authors declare that they have no competing interests.

## Authors' contributions

GL conceived of the study, participated in the experimental design and implementation, and revised the manuscript. SZ conceived of the study, supervised the theoretical design of the improved method, and revised the manuscript. XF participated in the experimental design and theoretical development of the method, carried out the implementation, and drafted the manuscript. All authors read and approved the final manuscript.
